# Tunable orbital angular momentum in high-harmonic generation

**DOI:** 10.1038/ncomms14971

**Published:** 2017-04-05

**Authors:** D. Gauthier, P. Rebernik Ribič, G. Adhikary, A. Camper, C. Chappuis, R. Cucini, L. F. DiMauro, G. Dovillaire, F. Frassetto, R. Géneaux, P. Miotti, L. Poletto, B. Ressel, C. Spezzani, M. Stupar, T. Ruchon, G. De Ninno

**Affiliations:** 1 Elettra-Sincrotrone Trieste, Area Science Park, 34149 Trieste, Italy; 2 Laboratory of Quantum Optics, University of Nova Gorica, 5001 Nova Gorica, Slovenia; 3 Department of Physics, The Ohio State University, Columbus, Ohio 43210, USA; 4 LIDYL, CEA, CNRS, Université Paris-Saclay, CEA Saclay, 91191 Gif-sur-Yvette, France; 5 Imagine Optic, 91400 Orsay, France; 6 Institute of Photonics and Nanotechnologies, CNR-IFN, 35131 Padova, Italy; 7 Laboratoire de Physique des Solides, Université Paris-Sud, CNRS-UMR 8502, 91405 Orsay, France

## Abstract

Optical vortices are currently one of the most intensively studied topics in optics. These light beams, which carry orbital angular momentum (OAM), have been successfully utilized in the visible and infrared in a wide variety of applications. Moving to shorter wavelengths may open up completely new research directions in the areas of optical physics and material characterization. Here, we report on the generation of extreme-ultraviolet optical vortices with femtosecond duration carrying a controllable amount of OAM. From a basic physics viewpoint, our results help to resolve key questions such as the conservation of angular momentum in highly nonlinear light–matter interactions, and the disentanglement and independent control of the intrinsic and extrinsic components of the photon's angular momentum at short-wavelengths. The methods developed here will allow testing some of the recently proposed concepts such as OAM-induced dichroism, magnetic switching in organic molecules and violation of dipolar selection rules in atoms.

## Abstract (web summary)

The controlled generation of extreme-ultraviolet beams with controllable topological charge has not been demonstrated. Here, Gauthier *et al*. report on the generation of extreme-ultraviolet optical vortices with femtosecond duration carrying a controllable amount of orbital angular momentum.

Angular momentum is a fundamental property of the photon, together with energy and linear momentum. In paraxial conditions, angular momentum may be split into an intrinsic part, the spin angular momentum and an extrinsic part called orbital angular momentum (OAM). Macroscopically, the OAM of light manifests itself in the spatial properties of the light beam and in particular in the shape of its wavefront. The most common light beams carrying OAM display Laguerre-Gaussian modes, which are solutions of the wave equation in the paraxial regime. They show an azimuthal phase dependence exp(−i*ℓφ*) (refs 1, 2), where *φ* is the azimuthal coordinate in the transverse plane and *ℓ*, called the topological charge, is indexing the mode. This phase shape creates a beam with a helical wavefront, a phase singularity in its centre and a donut-shaped intensity profile. A topological charge *ℓ* results in an OAM per photon equal to *ℓℏ* (ref. 1).

Laguerre-Gaussian modes, also called optical vortices, have been used in the visible range in many diverse applications. A photon pair can exhibit OAM entanglement^3^,^4^, which can be exploited in quantum cryptography. The donut-shaped intensity profile of an optical vortex allows bleaching the outer annular part of a sample and, applying stimulated emission depletion microscopy, allows to bypass the diffraction limit in optical microscopy^5^,^6^. Laguerre-Gaussian modes are also the tool of choice to manipulate particles^7^,^8^ or detect spinning objects^9^. Recently, increasing attention has been devoted to theoretical studies of fundamental interactions involving OAM beams in the extreme-ultraviolet (XUV) spectral range. For example, it was shown that OAM might be transferred to electronic degrees of freedom^10^,^11^. In ref. 12, it was also theoretically demonstrated that an XUV vortex can induce charge current loops in fullerenes with an associated orbital magnetic moment, which can be controlled by tuning the topological charge of the incident beam. These findings, if confirmed experimentally, could lead to new applications in magnetic switching using structured light.

These and other theoretical studies have sparked new experimental developments in the field of intense femtosecond XUV pulse generation. Schemes have been proposed for generating optical vortices using free-electron lasers^13^,^14^, which are still to be demonstrated. As a table-top and alternative to large-scale instruments, high-harmonic generation (HHG) is based on the frequency up-conversion of a high intensity femtosecond Vis-IR laser into the XUV range through a highly nonlinear process^15^. Recently, OAM has been studied in the context of HHG in gases. The first experimental work reported on the difficulty of generating optical vortices due to the non-conservation of the OAM during propagation in the gas jet^16^. Subsequent experiments, however, demonstrated the generation of optical vortices carrying a topological charge that is a multiple of the harmonic order^17^,^18^, in agreement with the expected conservation rule for a single driving beam^19^. Nevertheless, with such experimental schemes, the topological charge could not be tuned independently of the harmonic order. In addition, only high values of the topological charge could be obtained (except in ref. 16), while the controlled generation of XUV beams with low-order topological charge was not yet demonstrated. These two restrictions will severely limit the applicability of the above schemes in most of the recently proposed experiments.

In this article, we report on an alternative scheme to produce optical vortices carrying an arbitrary topological charge for any harmonic order using HHG. Our setup is based on a two-colour wave-mixing arrangement that combines a Gaussian with a frequency doubled Laguerre-Gaussian beam in a gas target, as proposed in ref. 17. To confirm that the generated donut-shaped high harmonics carry OAM we use a Hartmann sensor to measure their wavefronts. We demonstrate that the use of two driving beams allows efficient and robust generation of optical vortices with topological charges from *ℓ*=−1 to *ℓ*=4. Moreover, by exploiting the subtle physics principles on which the HHG process relies, we show how to favour the generation of a mode with a particular topological charge. This study provides an experimental verification of the conservation rule for OAM in HHG using two driving beams. Furthermore, our relatively simple setup makes HHG the first light source of femtosecond XUV pulses carrying a controllable amount of OAM.

## Results

### The experimental setup

**Figure 1:**
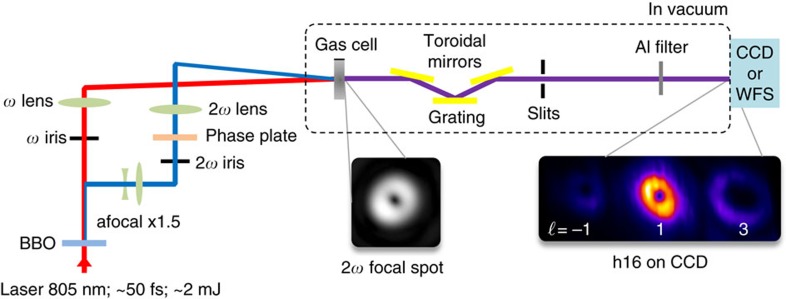
Sketch of the experimental setup. The collimated laser beam (8 mm at 1/*e*^2^) goes through a BBO crystal before entering a dichroic Mach-Zehnder interferometer in which the beam at 805 nm can be manipulated independently from the one generated after frequency doubling. The spiral phase plate converts the Gaussian beam into a Laguerre-Gaussian mode with *ℓ*_2*ω*_=1 in the second harmonic (2*ω*) arm of the interferometer, while the fundamental (*ω*) beam remains Gaussian. The mirrors and the delay line used to synchronize the two beams are not drawn, nor the half wave plate used to get identical linear polarizations. Both beams are focused with lenses of 75 cm focal length into the generation gas cell (≃1 mm length) filled with argon at a pressure of a few tens of mbar. To optimize the focusing geometry, both lenses are placed on translation stages, irises are positioned in both arms and a beam expander (× 1.5) is used in the 2*ω* arm. The XUV emission is sent into a monochromator (made of a grating between two toroidal mirrors and slits) before being imaged on an XUV CCD camera or a Hartmann wavefront sensor (WFS) placed in the far-field, ≃1 m downstream. (Insets) Typical intensity distributions at focus of the 2*ω* beam (left) and HHG vortices (right, labelled by their topological charge *ℓ*) generated at the 16th order and imaged on the CCD.

The experiment is sketched in Fig. 1 and was performed on the CITIUS light source, described in more details elsewhere^20^. A Ti:sapphire laser system (5 kHz repetition rate) provides pulses with a duration of ≃50 fs and energy of ≃2 mJ at a central wavelength of 805 nm. The pulses are sent through a 450 μm thick type-I BBO crystal, emitting the fundamental (*ω*) and second harmonic (2*ω*) beams. The two spectral components are then spatially separated in a Mach-Zehnder-like interferometer equipped with dichroic mirrors. The interferometer is used to manipulate individually the two beams before being focused by two independent lenses into a 1-mm long argon gas cell. In one arm of the interferometer, the 2*ω* beam is converted from a Gaussian to a Laguerre-Gaussian mode with *ℓ*_2*ω*_=1, using a spiral phase plate^21^. No OAM is imparted on the *ω* beam (*ℓ*_*ω*_=0). The crossing angle between the two beams in the gas cell can be adjusted by translating the last mirror of each arm. All modes generated at the frequency of one particular harmonic order of the fundamental frequency *ω* are isolated out from the other harmonic orders by a monochromator consisting of a grating placed between two toroidal mirrors, which provides an image of the HHG modes at the position of the slits located further downstream. The grating is used in the so-called conical geometry (diffraction in the vertical direction)^22^. The relatively low spectral resolution of the monochromator limits the spatio-spectral distortions (spatial chirp and pulse front tilt) of the imaged harmonic beam. Finally, the intensity distribution and the wavefront are measured in the far field using an XUV CCD or a Hartmann wavefront sensor (WFS) (developed by Imagine Optic, SOLEIL, and Laboratoire d'Optique Appliquée^23^).

### Measurement of the intensity profile

**Figure 2:**
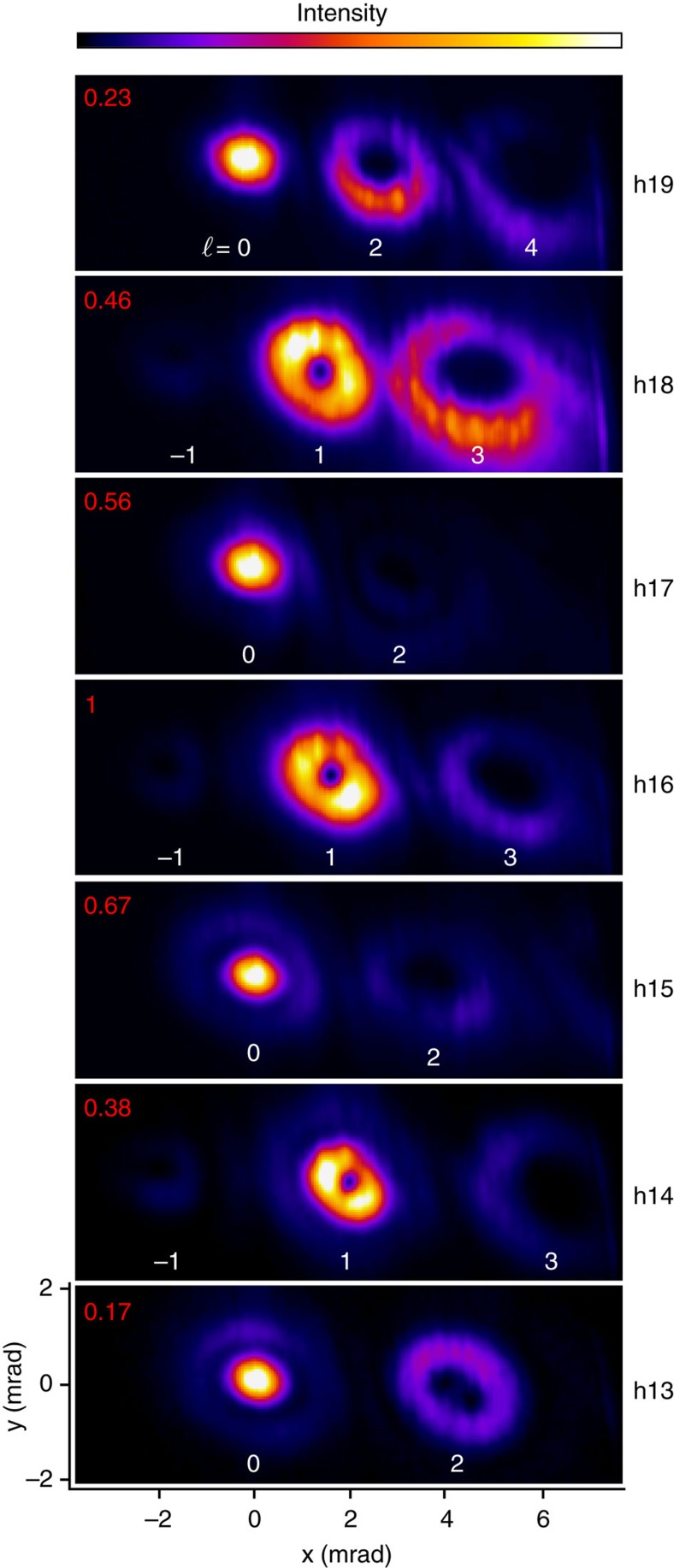
Far-field intensity patterns from the non-collinear wave-mixing scheme. Single-shot images for different harmonic orders were acquired using a CCD in identical generating conditions. Each mode is labelled by its topological charge *ℓ* that also corresponds to the number of absorbed 2*ω* photons. For better visualization, images for each harmonic order are shown with the full colour scale (colour bar on top). The integrated signal (top left corner) is normalized to h16. The number of photons incident on the CCD (after the metallic filter) for h16 was around 7 × 10^6^. Note that the angular acceptance of the monochromator (about 10 mrad) limited to three the number of modes that could be detected at once.

Figure 2 shows the far-field intensity profile of different harmonic orders obtained using an *ω*–2*ω* crossing angle of 13 mrad. The spatial axis origin is taken along the propagation axis of the *ω* beam. For each high-harmonic order, we observe the angular distribution (in the horizontal direction) of spatially separated modes. The emission angle of each mode is determined by the non-collinear phase-matching condition, in agreement with the conservation of energy, linear momentum and parity, previously established for HHG in the two-colour configuration^24^,^25^. It is convenient to use the photon picture to describe the possible pairs (*n*_1_, *n*_2_) that contribute to the emission of a given high-harmonic order *q* (with *q*=*n*_1_+2 *n*_2_), where *n*_1_ and *n*_2_ refer, respectively, to the number of photons absorbed from the *ω* and 2*ω* beams. In particular, parity requires that only the absorption of an odd total number of photons *n*=*n*_1_+*n*_2_ can lead to emission. Consequently, in Fig. 2, we observe modes generated from the pairs (19, 0), (15, 2) and (11, 4), contributing to the emission of photons with an energy corresponding to the 19th harmonic order (h19). For h18, the pairs experimentally observed are (20, −1), (16, 1) and (12, 3). Note that only the fundamental beam is contributing to the generation of the (*q*, 0) pair, while generation with the fundamental alone is not allowed for even harmonics. Positive and negative numbers in pairs are related, respectively, to sum- and difference-frequency generation.

The intensity patterns in Fig. 2 are consistent with the conservation of OAM, which has been confirmed by the phase front measurement reported below. This result is in agreement with the theoretical model developed in ref. 17, and demonstrates the transfer of OAM from the generating beams to high harmonics. For a two-colour wave mixing setup, we can generalize the conservation rule for OAM as:


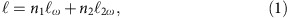


where *ℓ*, *ℓ*_*ω*_ and *ℓ*_2*ω*_ are the topological charge carried, respectively, by the high-harmonic, the *ω* and the 2*ω* beams. In the present experiment we focus on the case *ℓ*_*ω*_=0 and *ℓ*_2*ω*_=1, which gives simply *ℓ*=*n*_2_. Therefore, the topological charge carried by each mode is equal to the number of 2*ω* photons absorbed in the process. Consequently, except for pairs with *n*_2_=0, all modes display a ring-like intensity profile with zero intensity in the centre, characteristic of optical vortices. Moreover, for each harmonic order, the size of the rings increases with the number of absorbed 2*ω* photons, consistently with the increase of the topological charge, as demonstrated in ref. 18.

The results in Fig. 2 are representative of the main experimental finding for our generation conditions. For the low harmonic orders, in some cases, the modes in the far field are composed of inner and outer parts. Note that this feature exists for *ℓ*=0 with or without the presence of the 2*ω* driving beam. Such spatial profiles can be attributed to the contributions of two different quantum paths to the harmonic emission in combination with propagation effects^26^,^27^,^28^. The second noticeable feature is the spatial distortion of the mode profiles in the far field, which is due to aberrations in the XUV transport optics (monochromator) and to aberrations of the generating beams. Despite significant efforts to reduce such aberrations, a residual astigmatism was still present on the generating beams (visible in the focus of the 2*ω* beam, Fig. 1). Aberrations in the generating beams drive aberrations in the HHG emission, which are emphasized by the nonlinear response of the generation process. A residual effect related to this is visible in Fig. 2 at h13 for the *ℓ*=2 mode. When the modes are not fully spatially separated, the far-field intensity pattern also contains interference features.

### Measurement of the wavefront

**Figure 3:**
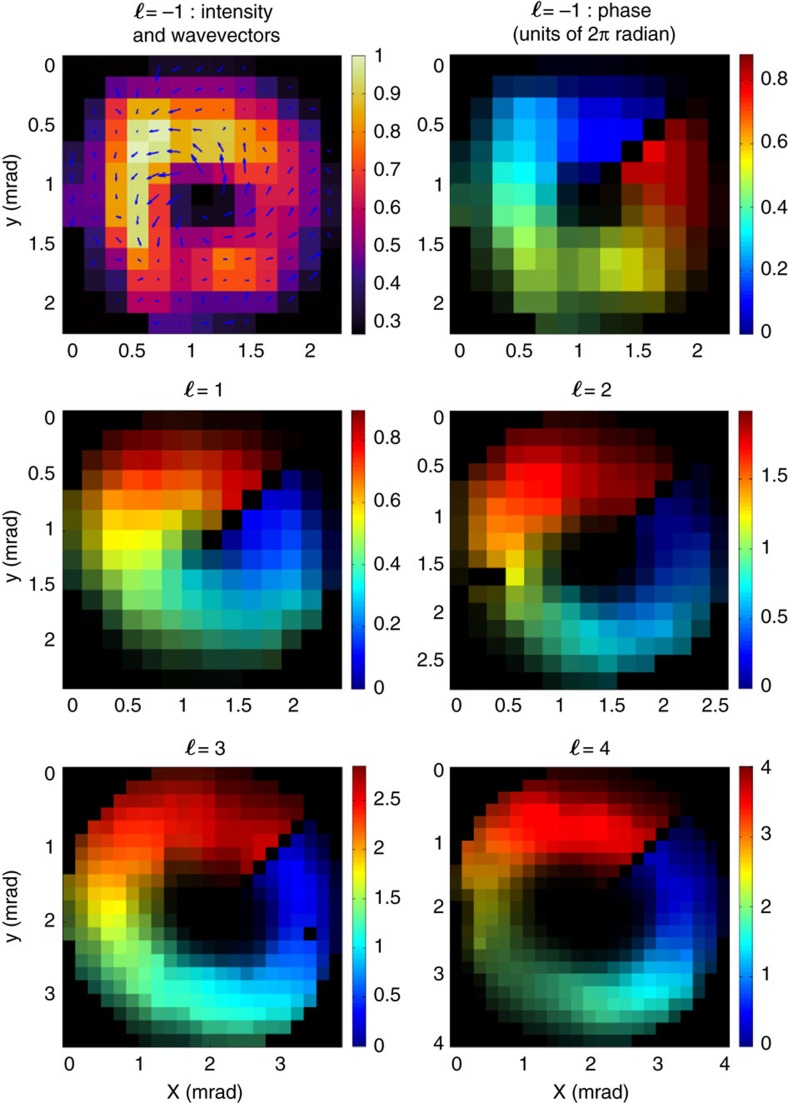
Intensity and wavefront measurements for several modes carrying various topological charges. The top-left panel shows the intensity (in arbitrary units) and the local wavevector (arrows) distribution for the *ℓ*=−1 mode of the 16th high-harmonic order emission (h16). The other panels show the measured wavefronts for *ℓ*=−1 and *ℓ*=1 for h16, *ℓ*=2 for h19, *ℓ*=3 for h18 and *ℓ*=4 for h19 (intensity and wavevector distribution for the latter cases is shown in Supplementary Fig. 1). The intensity is represented by the brightness and the phase (in units of 2*π* radian) by false colours. Black colour indicates pixels that contain no data (intensity lower than the detection threshold or pixels set to zero to enable wavefront reconstruction around the singularity). Note that the different panels have different colour scales. The yield of each individual mode was optimized (see next section), and a crossing angle of 15 mrad was used to guarantee a larger spatial separation between the modes, compared to the one in Fig. 2.

To confirm that the observed rings are actual (quasi) Laguerre-Gaussian modes, we performed wavefront measurements using a Hartmann WFS. Some representative results are displayed in Fig. 3. In the top-left panel, one can clearly recognize a spiralling wavevector around a singularity. The magnitude of the wavevector increases when approaching the centre of the beam. These two observations are the signature of an optical vortex. With our convention, we have a left-handed vortex for *ℓ*>0. The phase maps displayed in the other panels in Fig. 3 were obtained from the integration of the wavevector distribution^29^. To perform the integration around the singularity, we introduced a discontinuity in the data by setting it to zero on a line (at an arbitrary angle) going from the centre (singularity) to the edge of the image (additional information can be found in Supplementary Note 1). A reference wavefront (taken without the spiral phase plate) was used to correct for the phase aberrations due to the monochromator optics. We measured very smooth phases spiralling around the beam propagation axis going from 0 to about *ℓ* × 2*π* radians. The small discrepancies in the maximum variation of the phase across the map are attributed to a relatively low sampling of the wavefront. The reported measurements are a direct and unambiguous characterization of the OAM carried by the vortex beams, including its sign. They also demonstrate the possibility of directly measuring helical phase fronts in the XUV spectral range, which will be instrumental in the development of new generation light sources such as free-electron lasers carrying OAM.

### Yield of the harmonic vortices

**Figure 4:**
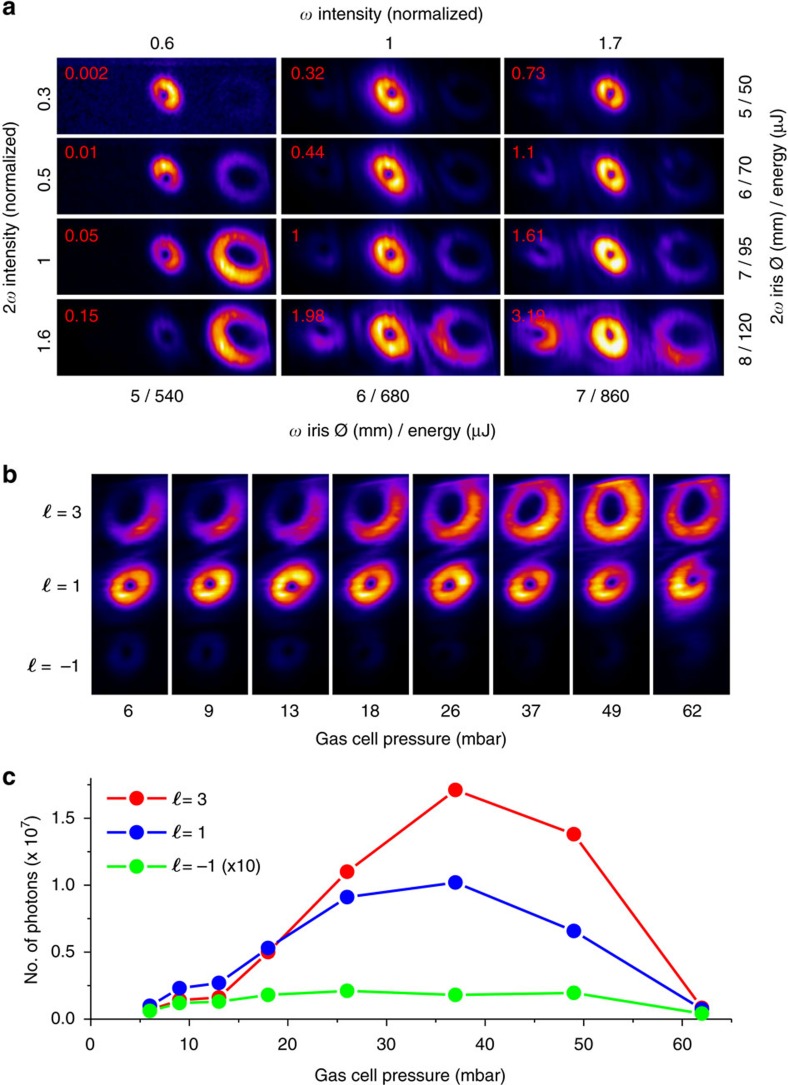
Yield of the modes as a function of the intensity and gas pressure for the 16th harmonic. (**a**) Evolution of the generated signal as a function of the intensity of the generating beams at focus. The intensity is normalized to an estimated intensity ratio *I*_2*ω*_*/I*_*ω*_ of about 18%, with *I*_*ω*_∼1.7 × 10^14^ W cm^−^^2^. The intensity is calculated from the measured iris aperture and transmitted energy. The signal is normalized to 1.7 × 10^7^ photons per shot on the CCD (top left corner of each image). (**b**,**c**) Evolution of the generated signal as a function of the argon pressure in the cell. (**c**) The curves display the integrated signal for each individual mode.

For a given high-harmonic order, the signal is not equally distributed between the modes. This is a known effect in highly non-linear wave mixing^25^,^30^, due to both the microscopic response of the medium and the macroscopic effects (propagation and phase matching) in HHG. This property can be exploited to optimize and favour the emission of a specific vortex by modifying the generation conditions. Figure 4 shows the evolution of the generated signal for three modes of the 16th harmonic order, when varying the iris aperture (that is, the transmitted energy) in each arm of the interferometer and the pressure in the gas cell. In Fig. 4a, the iris apertures modify the intensity as well as the size of both beams at focus. These parameters impact the individual atomic response in the gas jet (amplitude and phase of the dipoles), the phase matching conditions, and the propagation and reshaping of the fundamental beams in the medium^31^,^32^. The intensity ratio of the second harmonic to the fundamental beam (2*ω/ω*) was varied from a few percent up to 50%, spanning both perturbative and non-perturbative regimes. The relative intensity of the two colours, which affects the dipole amplitude, is the relevant parameter to explain the evolution of the signal within each mode^25^. This can be understood in terms of the probability that a certain pair (*n*_1_, *n*_2_) contributes to the emission of a given harmonic. A low 2*ω/ω* intensity ratio favours generation from pairs requiring a low *n*_2_ of photons absorbed from the 2*ω* field. On the other hand, a large intensity ratio 2*ω/ω* favours pairs with a high *n*_2_, particularly when the absolute intensity of the *ω* field is low. Figure 4b,c shows how the gas pressure impacts the yield of different modes. As expected, the yield increases for all modes when the pressure is increased. Surprisingly, at about 20 mbar the *ℓ*=3 mode overcomes the *ℓ*=1 mode. This feature cannot be explained by single atom effects. Including phase matching effects in the analysis of the experiment allows us to find the origin of this unexpected behaviour.

As noted in ref. 30, the peculiarity of the non-collinear scheme is that for an emission where *ℓ*=*n*_2_ 2*ω* photons are required, the phase matching equation must include an additional crossing term *k*_*∠*_(*ℓ*):


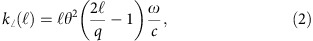


where *θ* is the crossing angle between the two driving beams and *c* is the speed of light. This term adds to the usual contributing terms: the geometrical phase advance, the dipole phase and the neutral and free electron dispersions. The sum of all these terms, Δ*k*, must be zero for the emission to be phase matched. Considering the cases *ℓ*=1 and *ℓ*=3, we obtain from the above equation 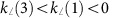. The quantities to be compensated are negative. For our experimental conditions the neutral dispersion, a positive quantity, is able to compensate for the non-collinear phase mismatch. Remarkably, as the dispersion of neutrals increases linearly with pressure, it may favour the emission of the *ℓ*=3 mode for high pressures.

**Figure 5:**
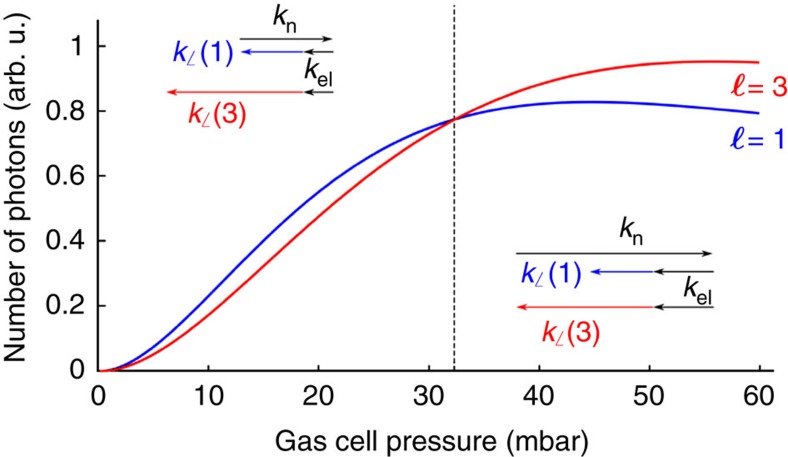
Calculated yield within the modes. The yield is calculated for the *ℓ*=1 (blue) and *ℓ*=3 (red) modes as a function of gas pressure (see Methods). The crossing of the two curves is indicated by the vertical dashed line. On the left (right) of this line, the phase matching favours the *ℓ*=1 (*ℓ*=3) mode, as shown in the insets. *k*_n_ and *k*_el_, are, respectively, the contributions of the neutral and free electron dispersion to the phase matching, and are the only terms that depend on the pressure.

In Fig. 5, using a simple model implemented with reasonable parameters (see Methods), we reproduce the main feature of Fig. 4c: a quadratic increase of the yield with pressure followed by a saturation at a few tens of millibars, and most remarkably, the overcome of *ℓ*=3 over *ℓ*=1 at about 30 mbar pressure. We note that the agreement is only semi-quantitative, as the crossing between the curves occurs at a pressure higher than the one observed experimentally. This can be attributed to uncertainties in several quantities used for the calculation (see Methods). Notice that the method used here describes the phase matching on-axis, that is, along the propagation axis of the HHG beams. It is independent of the spatial structures, and is not specific to vortex beams. Surprisingly, this effect seems to have stayed unnoticed in previous non-collinear HHG studies. In our case, it provides us with an additional knob to favour generation of harmonics with a particular topological charge. We note that, at the optimized gas pressure of 37 mbar, the *ℓ*=3 vortex has a flux of 8.5 × 10^10^ photons per second, which is comparable with some synchrotron beamlines in the same spectral range.

## Discussion

The results presented above enhance the capabilities of spatial shaping of HHG light compared to schemes which rely on a single generating beam^33^. Our method can be naturally extended to any combination of generating beams with various spatial properties. Similar wave-mixing schemes have been applied to the generation of circularly polarized high harmonics using two-colour Gaussian beams with various polarization states^34^,^35^. Combining one of these schemes with the method presented in this work will allow generation of femtosecond HHG pulses with independent control over orbital and spin angular momenta, paving the way towards new fundamental experiments in the field of light–matter interactions.

## Methods

### Model for the non-collinear HHG

We semi-quantitatively explain the observations of Fig. 4b by a simple model. If reshaping of the fundamental beam through nonlinear dispersion effects in the medium is neglected, the only phase matching terms evolving with pressure are the neutral and free electron dispersions, *k*_n_(*p*) and *k*_el_(*p*), respectively. For the sake of simplicity, we make the assumption that other terms do not depend on *ℓ*. With these assumptions, the phase mismatch as a function of the pressure *p* for each mode *ℓ* is:


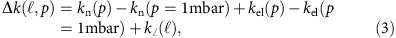


where we assume Δ*k*=0 for the *ℓ*=0 mode at a pressure of 1 mbar. The coherence length is then given by *L*_coh_=*π*/Δ*k*, while the absorption length is *L*_abs_=1/*σρ*, where *σ* is the argon cross-section and *ρ* is the atomic density. An estimate of the number of XUV photons for each mode can be calculated from these two quantities as^36^:


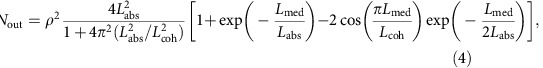


where *L*_med_=1 mm is the length of the medium. We evaluate this expression for the *ℓ*=1 and *ℓ*=3 modes for our experimental conditions. The atomic and free electron dispersions are calculated as in ref. 37 using an average degree of ionization of 4%. The model is only semi-quantitative due to uncertainties in several quantities, such as the level of ionization, the pressure at which Δ*k*(*ℓ*=0)=0, the measurement of the gas pressure in the interaction region, the effective length of the medium and the actual intensity at which HHG occurs. The predictions based on equations (3) and (4) are reported in Fig. 5.

### Data availability

All relevant data contained in this manuscript are available from the authors.

## Additional information

**How to cite this article:** Gauthier, D. *et al*. Tunable orbital angular momentum in high-harmonic generation. *Nat. Commun.* **8,** 14971 doi: 10.1038/ncomms14971 (2017).

**Publisher's note:** Springer Nature remains neutral with regard to jurisdictional claims in published maps and institutional affiliations.

## Supplementary Material

Supplementary Information Supplementary Figures and Supplementary Note
