# Supplementary material for: Tunable orbital angular momentum in high-harmonic generation
Source: Nat Commun. 2017 Apr 5;8:14971. doi: 10.1038/ncomms14971 (PMC5382323; doi:10.1038/ncomms14971)
Supplement: Supplementary Information — Supplementary Figures and Supplementary Note [file ncomms14971-s1.pdf]

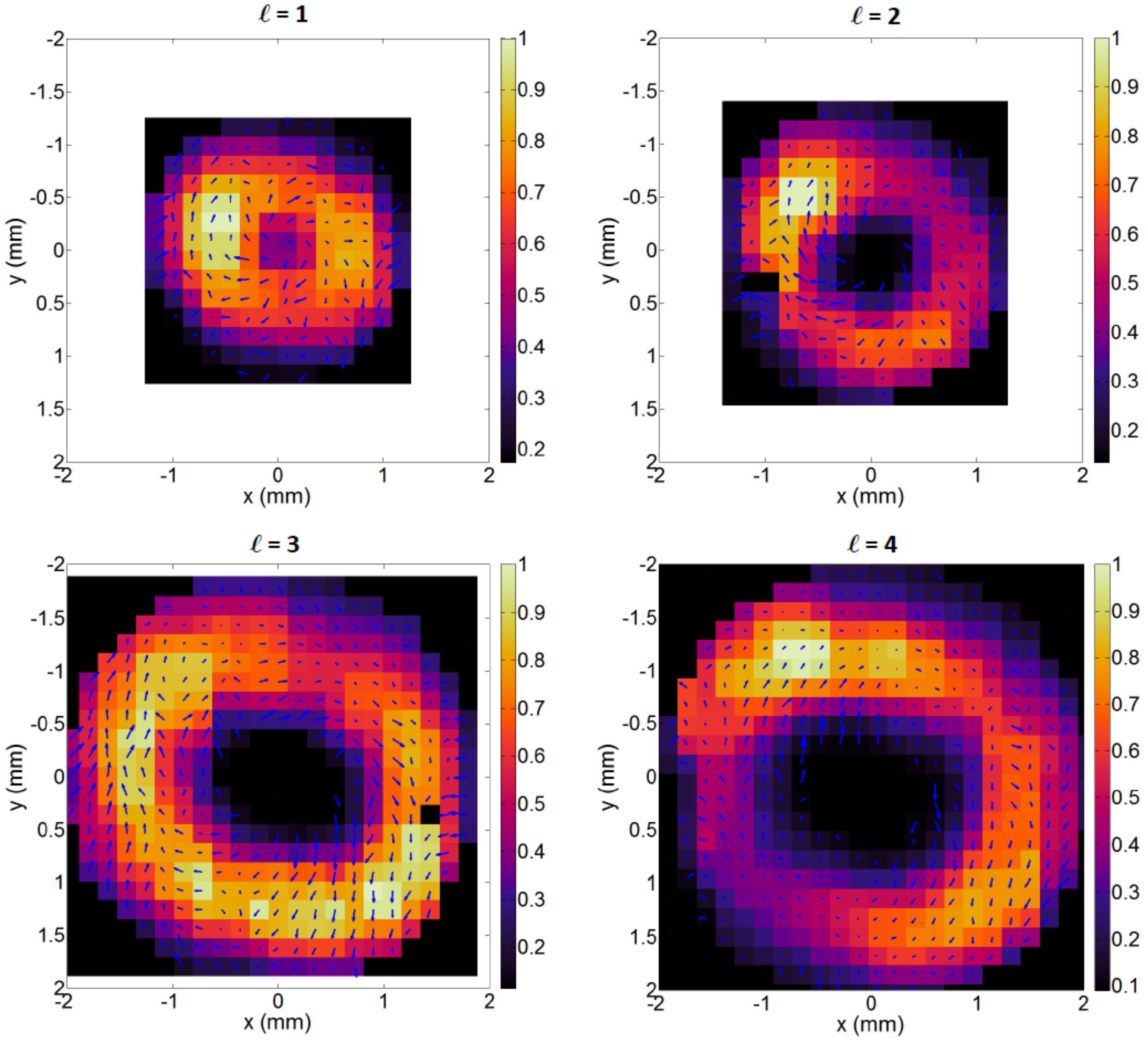

Supplementary Figure 1 | Wavevector distributions. Intensity (in arbitrary units) and the local wavevector (arrows) distribution for the modes from ℓ=1 to ℓ=4 shown in Figure 3 (see article).

Supplementary Note 1 | Details on the phase reconstruction.

The measurement and reconstruction of the phase was done with a commercial WFS from Imagine Optics. The reconstruction software is not programmed to detect and reconstruct phase singularities. In order to enable the integration of the phase, we created a discontinuity line in the wavevector measurements as explained in the article. No further data processing was required for reconstruction. The creation (on purpose) of a discontinuity line does not direct the reconstruction algorithm towards a solution with a helical phase structure. Moreover, a helical phase structure is not assumed during reconstruction. The following 2 figures show the reconstruction of the harmonic beam without the spiral phase plate on the 2ω driving beam (no OAM). In the Supplementary Figure 2, there is no discontinuity line, while the latter is present in the Supplementary Figure 3. As the panels show, there is no significant difference between the two reconstructions for a beam without phase singularity. Notice the presence of an aberration (coma) coming from the monochromator optics, which was corrected as explained in the article.

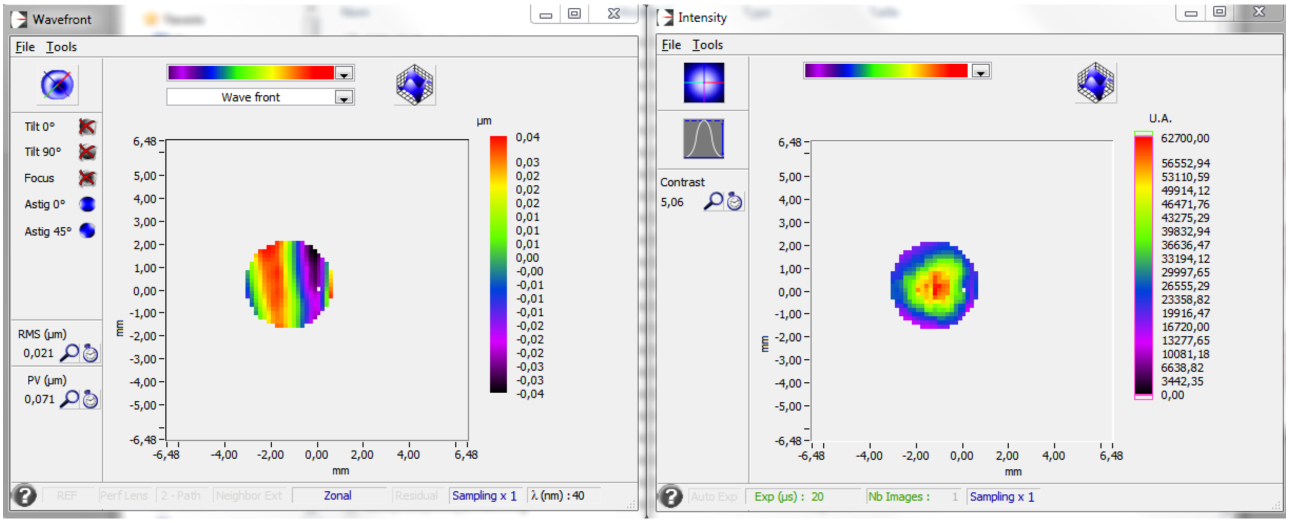

Supplementary Figure 2 | Screenshot of the WFS software showing the reconstructed wavefront and intensity in the absence of the discontinuity line for a HHG beam without OAM.

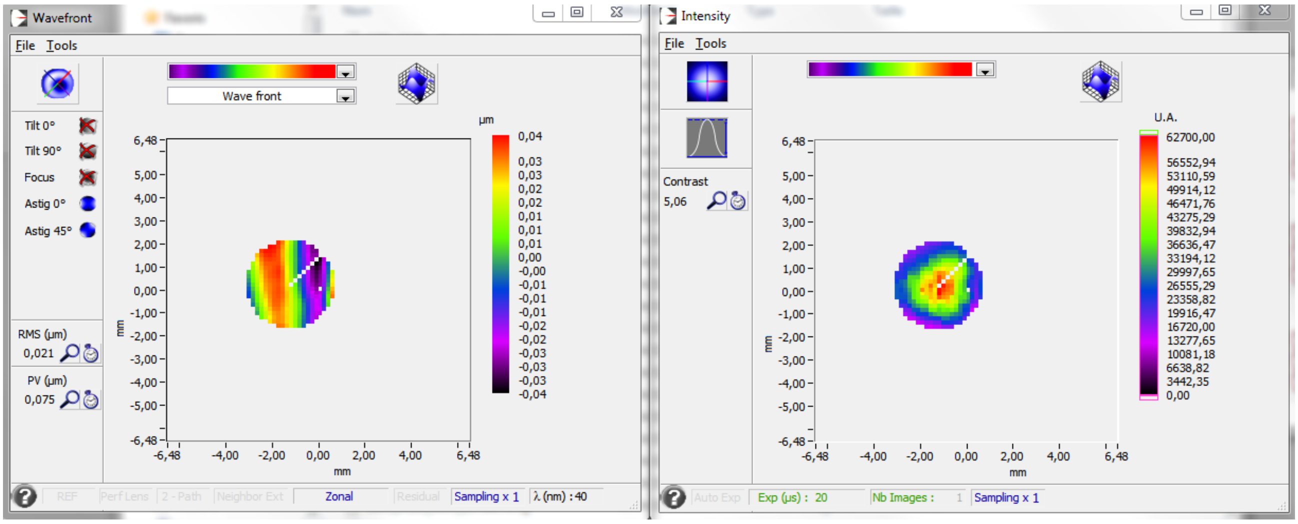

Supplementary Figure 3 | Screenshot of the WFS software showing the reconstructed wavefront and intensity in the presence of the discontinuity line for a HHG beam without OAM.
